# Interventions for improving employment outcomes for persons with autism spectrum disorders: A systematic review update

**DOI:** 10.1002/cl2.1185

**Published:** 2021-07-03

**Authors:** Carlton J. Fong, Joshua Taylor, Aynura Berdyyeva, Amanda M. McClelland, Kathleen M. Murphy, John D. Westbrook

**Affiliations:** ^1^ Texas State University San Marcos Texas USA; ^2^ Virginia Commonwealth University Richmond Virginia USA; ^3^ American Institutes for Research Austin Texas USA; ^4^ The University of Texas at Austin Austin Texas USA; ^5^ American Institutes for Research Austin Texas USA

## Abstract

**Background:**

The incidence of autism spectrum disorders (ASD) is on the rise. Currently, 1 in 59 children are identified with ASD in the United States. ASD refers to a range of neurological disorders that involve some degree of difficulty with communication and interpersonal relationships. The range of the spectrum for autism disorders is wide with those at the higher functioning end often able to lead relatively independent lives and complete academic programs even while demonstrating social awkwardness. Those at the lower functioning end of the autism spectrum often demonstrate physical limitations, may lack speech, and have the inability to relate socially with others. As persons with ASD age, options such as employment become increasingly important as a consideration for long‐term personal planning and quality of life. While many challenges exist for persons with ASD in obtaining and maintaining employment, some research shows that, with effective behavioral and social interventions, employment can occur. About 37% of individuals with ASD report having been employed for 12 months or more, 4 years after exiting high school. However, several studies show that individuals with ASD are more likely to lose their employment for behavioral and social interaction problems rather than their inability to perform assigned work tasks. Although Westbrook et al. (2012a, 2013, 2015) have reviewed the literature on interventions targeting employment for individuals with ASD, this review is outdated and does not account for recent developments in the field.

**Objectives:**

The objective of this review is to determine the effectiveness of employment interventions in securing and maintaining employment for adults and transition‐age youth with ASD, updating two reviews by Westbrook et al. (2012a, 2013).

**Search Methods:**

The comprehensive search strategy used to identify relevant studies included a review of 28 relevant electronic databases. Search terminology for each of the electronic databases was developed from available database thesauri. Appropriate synonyms were used to maximize the database search output. Several international databases were included among the 28 databases searched. In addition, the authors identified and reviewed gray literature through analysis of reference lists of relevant studies. Unpublished dissertations and theses were also identified through database searches. The programs of conferences held by associations and organizations relevant to ASD and employment were also searched. In sum, the search strategy replicated and expanded the prior search methods used by Westbrook et al. (2012a, 2013).

**Selection Criteria:**

Selection criteria consisted of an intervention evaluation using a randomized controlled trial or quasi‐experimental design, an employment outcome, and a population of individuals with ASD.

**Data Collection and Analysis:**

We updated the search from Westbrook et al., replicating and broadening the information retrieval processes. Our wide array of sources included electronic databases, gray literature, and conference and organization websites. Once all potentially relevant studies were located, pairs of coders evaluated the relevance of each title and abstract. Among the studies deemed potentially relevant, 278 were subjected to full‐text retrieval and screening by pairs of coders. Because many intervention studies did not include employment outcomes, only three studies met our inclusion criteria. Given the small number of included studies, meta‐analytic procedures were not used; rather, we opted to use more narrative and descriptive analysis to summarize the available evidence, including an assessment of risk of bias.

**Results:**

The systematic review update identified three studies that evaluated employment outcomes for interventions for individuals with ASD. All three studies identified in the review suggest that vocation‐focused programs may have positive impacts on the employment outcomes for individuals with ASD. Wehman et al. indicated that participants in Project SEARCH had higher employment rates than control participants at both 9‐month and 1‐year follow‐up time points. Adding autism spectrum disorder supports, Project SEARCH in Wehman et al.'s study also demonstrated higher employment rates for treatment participants than control participants at postgraduation, 3‐month follow‐up, and 12‐month follow‐up. Smith et al. found that virtual reality job interview training was able to increase the number of job offers treatment participants received compared to control participants.

**Authors' Conclusions:**

Given that prior reviews did not identify interventions with actual employment outcomes, the more recent emergence of evaluations of such programs is encouraging. This suggests that there is a growing body of evidence regarding interventions to enhance the employment outcomes for individuals with ASD but also greater need to conduct rigorous trials of vocation‐based interventions for individuals with ASD that measure employment outcomes.

## 1 BACKGROUND

### 1.1 The problem, condition or issue

#### 1.1.1 Background

According to the Centers for Disease Control and Prevention (CDC), the incidence rate of autism has increased over the past two decades to 1 in 59 children currently being identified with autism spectrum disorder (Baio et al., 2018). Internationally, autism spectrum disorders (ASD) prevalence is most recently reported at one in 160 children; however, this figure likely represents a significant underestimate (World Health Organization, 2019). Although individuals with ASD are capable of achieving academic and vocational success when provided with accommodations and support, outcomes for this group remain dire in terms of adult outcomes in employment and independent living (Roux et al., 2017; Wehman et al., 2018).

ASD refers to a range of neurological disorders that involve some degree of difficulty with communication and interpersonal relationships, as well as obsessive and repetitive behaviors. ASD occurs in all racial, ethnic, and socioeconomic groups. There is a wide range of effects demonstrated across the spectrum. Those at the lower functioning end of the spectrum often demonstrate physical limitations and may not be able to speak or relate socially to others. Those at the higher functioning end of the spectrum are often able to lead relatively independent lives, graduate from academic institutions, but may also be awkward in their social interactions and have difficulty developing friendships. The critical gap represented from the high to the low end of the spectrum dramatically affects employment‐related skills, abilities, and behaviors (CDC, 2011).

Given the increase of ASD prevalence and the number of students with ASD exiting public school settings, attention is increasingly focused on potential employment outcomes for individuals with ASD. Individuals without severe disabilities are eight times more likely to be employed than individuals with very severe disabilities (National Organization on Disability, 2000). Individuals with ASD are among those least likely to be employed (Dew & Alan, 2007; Roux et al., 2017); only 14%–15% of individuals diagnosed with ASD in the United States gained employment (Cameto et al., 2003; Roux et al., 2017).

Although economic conditions and employer attitudes affect employment opportunities, employment outcomes for individuals with ASD can be improved by providing social, behavioral, and work supports specific to individuals with ASD (Schaller & Yang, 2005; Wehman et al., 2013). Several studies have indicated that individuals with ASD are more likely to lose their employment for behavioral and social interaction reasons rather than their inability to perform work tasks (Bury et al., 2020; Dew & Alan, 2007; Hurlbutt & Chalmers, 2004; Solomon, 2020; Unger, 1999; Wilczynski et al., 2013). Conversely, specific employer practices—sometimes referred to as demand side strategies—have been linked to improved outcomes for employees with autism such as collegial understanding, positive work environment, and provision of accommodations (Chan et al., 2010; Hayward et al., 2019). Employment support service providers must often provide social and behavioral interventions along with other supports to effectively facilitate obtaining and maintaining employment for individuals with ASD (Ham et al., 2014). However, relatively few employment support service providers including state vocational rehabilitation counselors have an in‐depth understanding of services that are associated with developing successful employment outcomes for individuals with ASD (Dew & Alan, 2007). Social skills and behavioral interventions designed specifically for workplace settings have received increased attention in the research literature as a means of addressing this barrier to employment for individuals with ASD (Gorenstein et al., 2020).

Each state in the United States has at least one state agency that is charged with the provision of vocational rehabilitation services to facilitate employment outcomes for eligible individuals with disabilities. Data accumulated through the national network of state vocational rehabilitation agencies suggest that few individuals with autism requested services. Of the individuals who did request services, few were successfully employed as an outcome of the service. However, in its latest report, the National Vocational Rehabilitation Service System indicated that in the 2007 fiscal year, it provided services that successfully placed 1774 individuals with ASD into employment situations that continued for 90 or more days (Rehabilitation Services Administration, 2009).

### 1.2 The intervention

Interventions designed to improve the employment outcomes for individuals with ASD are varied and multifaceted. Interventions can be broadly categorized as aiming to improve access to employment or success in their employment settings, or both within a comprehensive intervention package such as supported employment or customized employment (Baker‐Ericzén et al., 2018; Smith et al., 2019; Wehman et al., 2018). Across the developmental spectrum, these interventions may target individuals ranging from those transitioning from secondary or postsecondary schooling to the workforce or individuals maintaining their employment status. Programs are often government‐funded and provided in conjunction with vocational rehabilitation efforts targeted toward individuals with disabilities.

### 1.3 How the intervention might work

Employment‐related interventions for individuals with ASD can function in different ways. For instance, several employment interventions for individuals with autism include job development services such as job searching, application preparation, interview skills, and job negotiation (Wehman et al., 2018). As individuals with ASD may possess varying levels of social competencies, interventions that enhance social skills can be particularly effective for job‐related activities such as interviewing and interpersonal interactions with other employees (Grob et al., 2019). Although many individuals with autism are currently served in segregated prevocational settings, previous research has indicated that individuals with previous experience in these segregated settings are actually less likely to eventually gain competitive integrated employment (Taylor et al., 2021). In addition to job development services, another set of interventions often targets work‐related skills such as specific job training tasks that may help individuals with ASD maintain their employment through on‐the‐job training and long‐term support (Schall et al., 2020). Thus, we hypothesize that the intervention might work to address job development before employment or on‐the‐job skills and thus improve gainful employment outcomes.

### 1.4 Why it is important to do the review

This current systematic review is an update to two prior Campbell reviews on this topic, one focused on employment‐related interventions for adults (Westbrook et al., 2012a) and the other for youth (Westbrook et al., 2013). Thus, research has been conducted in the area of autism and employment, including prior systematic reviews. However, the landscape of evidence has changed since these systematic reviews have been conducted, and an updated systematic review on the effectiveness of adult employment assistance interventions for individuals with ASD would not only demonstrate the extent and magnitude of more recent interventions' effects, but also provide ideas for further research that can inform implementation and refinement of related employment‐focused programs.

Additionally, several literature reviews have been published in the peer‐reviewed literature related to the employment‐related interventions for individuals with ASD (e.g., Bennett & Dukes, 2013, Hendricks, 2010; Jacob et al., 2015; Rashid et al., 2018; Schall et al., 2020). These recent reviews have provided important and specific findings related to such topics as the employment of individuals with ASD through K‐12 to work transition supports (Bennett & Dukes, 2013), the economic costs and benefits of employing individuals with ASD (Jacob et al., 2015), common challenges and related strategies (Hendricks, 2010), and employer practice interventions (Rashid et al., 2018). Likewise, Schall et al. (2020) incorporated a scoping review methodology designed to provide a broader review of related interventions to promote the employment of individuals with ASD. Thus, there is a need for an updated systematic review directly focused on the effectiveness of adult employment assistance interventions for individuals with ASD.

This review intends to identify and describe the effectiveness of behavioral and social interventions that prepare individuals with ASD for employment. In addition, the review intends to serve as guidance for planners of transition and adult programs and as an indicator of how further research would be beneficial.

More needs to be known about the effectiveness of strategies that are successful in supporting the transition of youth and adults with ASD into employment settings. As the population of persons with ASD grows, more demands and expectations will center on schools and vocational support agencies to effectively facilitate their transition into appropriate work and community living settings. Although prior reviews on this topic have emerged since the Westbrook et al. (2012a, 2013) reviews, a focus on the best‐quality evidence and on specific employment outcomes is still needed (e.g., Hedley et al., 2017).

## 2 OBJECTIVES

To determine the effectiveness of employment assistance in securing and maintaining employment for adults and transition‐age youth with ASD.

## 3 METHODS

### 3.1 Criteria for considering studies for this review

A two‐stage process was used to determine inclusion or exclusion of studies: (1) title and abstract stage and (2) full‐text stage.

#### 3.1.1 Title and abstract stage

With Endnote software, two coders independently assessed and selected for advancement to the next stage of inclusion if titles and abstracts met at least one of the two following criteria:

##### Participants

The participant sample of study included individuals of any age with a diagnosis of ASD.

##### Intervention

The focus of the study intervention centered on the topic of employment. The types of employment included were competitive, supported, or integrated employment. Studies in which the experimental groups assigned to sheltered work or nonintegrated work interventions were excluded from the review due to not providing the integrated or mainstream format of employment. Studies that reported effect sizes were included in the review. If these criteria were not clear from the title or abstract, the study was advanced for retrieval of the full text to determine eligibility.

#### Full‐text stage

Full‐texts of studies from all citations/abstracts advanced from Stage 1 were retrieved for a final determination of inclusion in the review and analysis. All of the following criteria were required for each study to be included in the review and analysis. Two coders independently assessed study inclusion at this stage.

##### Research design

Studies used an experimental or randomized controlled trial (RCT) design or quasi‐experimental design to report the effects of the intervention.

##### Participants

The recipients of the intervention were individuals of any age with an ASD and were voluntarily seeking assistance in obtaining employment. Study participants with ASD eligible for inclusion were individuals with Asperger syndrome, autism, Rhett syndrome, childhood disintegrative disorder, or pervasive developmental disorder‐not otherwise specified, as defined in the DSM‐IV‐TR and diagnosed by an appropriate professional. Participants not employed at the time of the study intervention were the focus of this review. Reviewers did not exclude studies in which the participant pool included both participants who had an employment history and those who did not. Individuals who were employed before an intervention study were not excluded in this review. Study participants with ASD and other secondary disabilities were included; however, study participants with sole disabilities such as mental retardation, schizophrenia, attention deficits, or other nonautism related conditions were not included.

##### Intervention

The interventions were required to provide adult employment assistance intended to produce employment outcomes for individuals with ASD. The interventions needed to address social, behavioral, and/or cognitive dimensions related to the acquisition and maintenance of employment among the study participants. The interventions also needed to involve relatively specific and structured experiences designed to support employment placement: for example, providing guidance in completion of applications, resumes, and engaging in interviews; shaping of work skills and appropriate employment setting social skills; employment site supports; designing of jobs/tasks around the expressed needs and desires of participants; teaching of work‐related communication skills; or working directly with employers in the structuring of work and work setting features for individuals with ASD. Employment assistance interventions of any duration were included providing that the study authors supplied an adequate description.

##### Outcome measures

Eligible outcomes included subsequent attainment of an employment placement and specific data about the duration and/or retention of that placement must have been provided. Gainful employment included competitive, integrated, or supported employment. Sheltered work or nonintegrated work was not considered as an outcome measure for this review. Employment encompassed full or part‐time placements.

##### Publication status

Published and unpublished studies were eligible for inclusion in the evidence pool.

##### Country of origin and language of publication

Studies that were conducted in any country were eligible. We did not exclude studies reported in languages other than English. Although we did not specifically search for non‐English literature, we did search selected international databases. A translator assisted the reviewers to determine whether non‐English studies that were retrieved should be included in the full‐text review. Specifically, a few articles in Japanese were reviewed for relevance but ultimately excluded when they did not satisfy exclusion criteria.

### 3.2 Search methods for identification of studies

#### 3.2.1 Electronic searches

We searched within a large number of electronic databases using search terms from domains associated with ASD, employment, and intervention terminology. Due to the combining of the Westbrook et al. (2012a, 2013) systematic reviews, we did not place any age‐related terms in our search strategy. In addition, we searched for our articles after 2008, the most recent year the Westbrook et al. reviews concluded their searches. Our electronic searches concluded in August 2017.

Using three domains (population, treatment, and domain), all linked by appropriate Boolean terms, we used the following search terms (* for truncation):

POPULATION

Autis*

Asperger*

ASD

Pervasive developmental disorder*

Pervasive development disorder*

Pervasive child development disorder*

PDD‐NOS

Rett*

Childhood disintegrative disorder*

Heller's syndrome*

Heller syndrome*

DOMAIN

employ*

pre‐employ*

work*

vocation*

occupation*

trade*

career*

job*

rehabilitat*

skill*

We searched the following databases:

- ABI Inform Global
- Academic One Premier (Gale)
- Business Source Complete
- Canadian Research Institute
- CBCA
- CINAHL
- Cochrane Library
- Dissertations Global and Theses
- Education Source
- EPPI‐Centre
- ERIC
- Family Studies Abstracts
- FRANCIS
- LearnTechLib
- Psychological and Behavioral Sciences Collection (PBSC)
- PsycCritiques
- PsycINFO
- PubMed
- REHABDATA
- SCIE
- Science & Technology
- SCOPUS
- Social Care Online
- SocINDEX
- Web of Science Core Collection
- WorldCat

#### 3.2.2 Searching other resources

Gray literature identified through electronic searches were submitted to the same inclusion criteria as other studies. The same time range (2008–2017) for gray literature types of studies was specified as the other studies. References from individual studies were searched for potential studies to consider for inclusion. In addition, unpublished dissertations and theses were identified through the search strategy for review and consideration. Also, recent conference proceedings from relevant associations and conferences were reviewed to identify unpublished studies to include in the review:

Autism Society of America

National Association of Rehabilitation Research and Training Centers

International Society for Autism Research

National Alliance for Autism Research

Autism Research Institute

National Autistic Society (UK)

Autism Research Centre (Cambridge)

Conference proceedings that were reviewed included:

Asia Pacific Autism Conference 2009

PENN Autism Network Conference.

Autism Society National Conference

NARRTC Annual Conference

Annual International Meeting of Autism Research (IMFAR)

National Autistic Society's Professional Conference

Cambridge Autism Research Conference

### 3.3 Data collection and analysis

#### 3.3.1 Selection of studies

Studies were screened for inclusion/exclusion decisions at two stages, Stage 1: citation and abstract and Stage 2: full‐text. The same two coders served as independent reviewers at both stages. A third party was not needed to resolve a coding value difference.

#### 3.3.2 Citation and abstract stage

At Stage 1, the decision for advancing the retrieved citations and abstracts in Endnote to the full‐text stage retrieval was made independently by both reviewers based on meeting two items from a and b of the following questions or a designation by a reviewer of “unsure” (item c):

A. Are the participants identified, described, and defined under the Autism Spectrum Disorder category?
B. Is this abstract/citation about employment?
C. Unsure of meeting inclusion criteria?

If the reviewers were “unsure,” the citation/abstract was advanced to the full‐text stage for a final inclusion decision. Inter‐rater reliability was tested on a sample of 25 studies at this stage and was found to be 95%. Coding differences were resolved through discussion between pairs of reviewers.

#### 3.3.3 Full‐text level

At the full‐text Stage 2 level, full texts of all citations advanced from Stage 1 were obtained and coded for an inclusion/exclusion decision. Two reviewers for each study independently decided whether to include the retrieved full‐text studies in the final analyses. An inclusion decision for advancement to the coding stage of the process required that a study met all the criteria presented earlier. When multiple studies used the same sample or outcome data, the study providing the most complete information focusing on our desired intervention outcome was selected for inclusion. Inter‐rater reliability was established before initiating coding activities, minimizing coding disagreements. At the Full‐Text Stage 2 level, the two reviewers recorded all excluded studies and the reason for exclusion independently. The two reviewers resolved their differences through discussion until they came to agreement.

#### 3.3.4 Data extraction and management

Data that were extracted and coded from the primary studies included: publication source, subject characteristics, sample source, employment setting, intervention characteristics, type of employment, and outcome measurement. See Supporting Information Appendix A for a copy of the coding form.

Coding was completed by two independent coders with an Excel‐based coding sheet. Any discrepancies were resolved by a third‐party author.

#### 3.3.5 Assessment of risk of bias in included studies

Included studies were coded by two independent reviewers for methodological quality addressing dimensions that included:

- Design type
  o RCT individual randomized design
  o RCT group randomized design
  o Quasi‐experiment: equivalent comparison design (individuals)
  o Quasi‐experiment: equivalent comparison design (groups)
  o Quasi‐experiment: nonequivalent comparison design (individuals)
  o Quasi‐experiment: nonequivalent comparison design (groups)
  o Quasi‐experiment: regression discontinuity

- Unit of assignment (e.g., individual vs. group/class)
- Unit of analysis (e.g., intention to treat, test only, treated)
- Attrition from pretest to posttest
- Fidelity of implementation (e.g., following replicable program of intervention)
- Blinding of assessors/interventionists

In addition, an evaluation of the potential risk of bias of all included studies was conducted using “Risk of Bias” procedures developed by Higgins and Green (2011) in which studies are evaluated across five sources of potential bias. A report of this analysis is provided in Section Selection Criteria, 4.

#### 3.3.6 Measures of treatment effect

The magnitude of the intervention effects was calculated as either a Cohen's *d* or an odds ratio (OR), depending on the nature of the outcome. If the outcome was continuous (i.e., amount of wages earned), the effect size was calculated as a Cohen's *d*, or standardized mean difference. When the outcome was dichotomous (i.e., employed or not), then we calculated ORs for effect sizes. Effect sizes were calculated directly from reported means and standard deviations or percentages for the experimental and control groups for studies that reported such statistics along with the sample sizes.

#### 3.3.7 Unit of analysis issues

When studies reported multiple outcomes for the same group comparison, such as various timepoints, there are dependency issues that may arise when synthesizing effect sizes together. Because we did not statistically integrate any effects together, but rather discussed narratively the individual effects, issues of dependency do not impact the results of our summary.

#### 3.3.8 Dealing with missing data

After using our coding guide to extract data, there were instances of missing data where studies did not report complete information covered in the coding guide. Because effect size data were complete, and no moderator analyses were conducted, we did not attempt to request information from authors or conduct any imputation techniques.

#### 3.3.9 Assessment of heterogeneity

Because of our narrative approach to describe interventions effects given the small number of included studies, we did not perform a formal assessment of heterogeneity.

#### 3.3.10 Assessment of reporting biases

Due to the small number of studies, we also did not explore any selection or reporting biases.

#### 3.3.11 Data synthesis

Due to the small number of included studies, we opted to describe the findings of the included studies narratively. We conducted no quantitative data synthesis of the effects.

#### 3.3.12 Subgroup analysis and investigation of heterogeneity

We did not conduct any subgroup or investigations of heterogeneity through moderator tests.

#### 3.3.13 Sensitivity analysis

We did not conduct any sensitivity analyses.

## 4 RESULTS

### 4.1 Description of studies

#### 4.1.1 Results of the search

The results of the search yielded a total of 12,982 citations published after 2008. Upon double‐screening all titles and abstracts, 278 citations remained for further examination of their full‐text documents. After double‐screening all full‐text documents, three studies were included.

#### 4.1.2 Included studies

This section narratively summarizes the included studies and their characteristics such as research design, sample sizes, setting, recruitment, participants, interventions, and outcomes. Note that the studies will be referred to by the last name of the first author and the year of the publication.

#### 4.1.3 Research design

All three included studies used RCT designs (Smith et al., 2015; Wehman et al., 2014, 2017). Two of the three studies used a random number generator to assign individual participants to treatment or control groups (Wehman et al., 2014, 2017). No studies used stratification or wait‐listed control groups as part of their design. All three studies included data from follow‐up points.

#### 4.1.4 Sample sizes

The unique number of participants from both treatments and comparison groups across the three included studies was 108. The average sample size per study was 36 participants (T: 23.3 participants; C: 12.7 participants).

#### 4.1.5 Setting and recruitment

In two of the three studies, local education agencies (LEAs) assisted in the recruitment of eligible participants meeting inclusion criteria (Wehman et al., 2014, 2017). Recruitment in the remaining study was conducted through advertisements at local universities, community‐based service providers and support groups, and online support groups (Smith et al., 2015).

The settings for the treatment groups in Wehman et al. (2014, 2017) were located at community‐based employment sites at hospitals within a single region. The control groups for these studies attended high school and continued their normal course of study for the duration of the academic year. The setting in the third study was private offices for the intervention and a clinical space for the assessment phase (Smith et al., 2015).

#### 4.1.6 Participants

##### Disability type

None of the three studies included specific diagnostic disability assessment as part of their design, instead accepting previous medical and educational diagnoses. Since all three studies were conducted shortly after publication of the Diagnostic and Statistical Manual of Mental Disorders Fifth Edition, previously used differential ASD labels were reported in each study. One study reported that all participants had diagnoses of high‐functioning autism (Smith et al., 2015). Two other studies reported specific autism diagnoses with Wehman et al. (2014) reporting treatment group composed of 62.5% autism; 25% PDD‐NOS; 12.15% Asperger's, and control group of 81% autism; 12.15% PDD‐NOS; 6.3% Asperger's. Wehman et al. (2017) reported that the treatment group consisted of 70.9% autism; 19.4% PDD‐NOS; 9.7% Asperger's syndrome, and the control group 72.2% autism; 33.3% PDD‐NOS; 5.5% Asperger's syndrome.

##### Age and ethnicity

All studies reported both the age and race/ethnicity of participants in both treatment and control groups. Mean ages of participants ranged from 19.13 years (Wehman et al., 2014) to 25.0 years (Smith et al., 2015). Of the total participants included in this review, 50% reported race/ethnicity as White, followed by 42% African American, 4% Latino, and 3% Asian.

##### Education level and socioeconomic status

Two of the three studies used participants who were in their last year of high school (Wehman et al., 2014, 2017). Smith et al. (2015) reported parental education, but not participant education level. None of the studies mentioned socioeconomic status, though one study included information about the number of parents in the household and parents' occupation type (Wehman et al., 2017).

##### Interventions

Interventions were identical in two of the three studies. Both Wehman et al. (2014) and Wehman et al. (2017) used the Project SEARCH Plus ASD Supports (PS‐ASD) model. The PS‐ASD intervention consists of 900 h embedded in a community‐integrated business setting. Classroom instruction at the business comprises 180 h, along with an additional 720 h of internship experience. PS‐ASD is a collaborative model between students with autism, their family members, a LEA, a local community rehabilitation program, and a local vocational rehabilitation agency. Staffing and funding for a teacher, assistants, and job coaching services are provided by various stakeholders in this collaboration. PS‐ASD adds an additional component to traditional Project SEARCH models to include specific strategies and staff expertise specific to autism based on the principles of Applied Behavior Analysis.

In the third study, Virtual Reality Job Interview Training (VR‐JIT) was used (Smith et al., 2015), which consisted of a digital simulation of a job interview with a human resources representative from a department store. While the application can be accessed through a typical computer, in the study, participants accessed it using wearable virtual reality headsets.

##### Outcomes

Employment status was the primary outcome measure of each of the three included studies (Smith et al., 2015; Wehman et al., 2014, 2017). Smith et al. (2015) also included outcome measures specific to job searching and interviewing such as time spent looking for a job and number of interviews completed.

#### 4.1.7 Excluded studies

Studies were excluded on the following bases: (1) did not include an employment outcome; (2) did not assess the impact of an intervention; (3) did not include a sample of individuals with ASD.

### 4.2 Characteristics and risk of bias in included studies

In the following sections, we describe the characters of the three included studies, outlining the method, setting, participant, intervention, and outcome. We also include the risk of bias assessment.

***Wehman et al.*** (***2014***)

*Characteristics of study*

**Table:** 

Method	Randomized controlled trial
Setting	Community business (Hospital)
Participant	T: 24 participants (75% male); mean age 19.96C: 16 participants (68% male); mean age 19.13T: 62.5% autism; 25% PDD‐NOS; 12.15% Asperger'sC: 81% autism; 12.15% PDD‐NOS; 6.3% Asperger's
Intervention	Description: Project SEARCH is a 9‐month internship model where youth with developmental disabilities in their last year of high school are embedded in a large community business. Students with developmental disabilities who participated in this model rotated through three 10–12 week internships within the business. Sessions: 900 total hours over 9 months (720 h internship; 180 h of classroom at business)
Outcome	Work status; wages

Risk of bias

**Table:** 

Bias	Authors' judgment	Description
Selection bias	Unlikely	Random assignment conducted by numbering participant applications and using a random number generator to assign to treatment or control. A researcher without connection to the study conducted the randomization
Performance bias	Very likely	No discussion of researcher blinding in report
Detection bias	Very likely	No discussion of assessor blinding in report
Attrition bias	Uncertain	Attrition occurred following randomization with four participants exiting the control group before data collection. No attrition reported from the treatment group. Since control group participants exited before data collection, it is unclear whether there was attrition
Reporting bias	Unlikely	Reporting complete for work status, supports intensity scale for behavioral and medical supports, and reported use of psychotropic medication
Other bias	Likely	Treatment group participants were located at two hospitals in the same region with largely the same staff at each site. Authors noted possible bias as a result of geographic location and staff skill and training

**Smith et al.** (**2015**)

*Characteristics of study*

**Table:** 

Method	Randomized controlled trial
Setting	Not reported
Participant	T: 15 participants (73.3% male); mean age 25C: 8 participants (75% male); mean age 23.1Additional participant details: High‐functioning autism
Intervention	Description: Virtual Reality Job Interview Training (VR‐JIT) is an intervention to improve job interview skills for adultswith a range of disabilities. Trainees simulated a job interview with a virtual human resources representative at a large department store. Session: Six‐month program
Outcome	Role‐play performance; VR‐JIT performance; work status

*Risk of bias*

**Table:** 

Bias	Authors' judgment	Description
Selection bias	Very likely	No discussion of randomization and group assignment procedures
Performance bias	Very likely	No discussion of researcher blinding
Detection bias	Very likely	No discussion of assessor blinding
Attrition bias	Uncertain	No discussion of participant attrition
Reporting bias	Uncertain	Reporting complete for demographics, vocational history, cognition, social responsiveness, and employment at follow‐up; no discussion of missing data
Other bias	Likely	Small sample size and limited measurement of participant covariates led to a large amount of unexplained variance in data as evidenced by a marginal *p*‐value of 0.048 for null hypothesis [JPT3]

**Wehman et al.** (**2017**)

*Characteristics of study*

**Table:** 

Method	Randomized controlled trial
Setting	Community business (Hospital)
Participant	T: 31 participants (77.4% male); mean age 19.5 C: 14 participants (61.1% male); mean age 19.7 Severity levelT: 70.9% autism; 19.4% PDD‐NOS; 9.7% Asperger's C: 72.2% autism; 33.3% PDD‐NOS; 5.5% Asperger's
Intervention	Description: Project SEARCH model was modified by adding additional supports for individuals with ASD. SEARCH increased the structure and intensity of the learning experiences by ensuring the use of applied behavior analytic (ABA) techniques, in addition to increasing the specific social communication skills needed for success at work. Sessions: 900 total hours over 9 months (720 h internship; 180 h of classroom at business)
Outcome	Work status; wages

*Risk of bias*

**Table:** 

Bias	Authors' judgment	Description
Selection bias	Very likely	No discussion of specific randomization process and selection method other than that the method was not a waitlist design
Performance bias	Very likely	Researchers reported that blinding was not possible due to the nature of the data collection and analysis procedure
Detection bias	Very likely	Researchers reported that blinding was not possible due to the nature of the data collection and analysis procedure
Attrition bias	Likely	Attrition occurred between group assignment and outcome time point. Five participants originally assigned to the control group elected to reapply for randomization after 1 year; four of the five were reassigned to the treatment group
Reporting bias	Unlikely	Reporting complete for work status, supports intensity scale for behavioral and medical supports, demographic information, prior unpaid and paid work experience, and reported use of psychotropic medication
Other bias	Likely	Treatment group participants were located at one site with largely the same staff. Authors noted possible bias as a result of geographic location and staff skill and training

### 4.3 Synthesis of results

Due to the few studies included in the update, we narratively describe the effects of the three included studies. Two studies (Wehman et al., 2014, 2017) demonstrated that Project SEARCH was effective to increase employment rates for youth with ASD. Specifically, Wehman et al. (2014) found that 21 out of 24 treatment participants were employed compared to 1 out of 16 in the control group after 9 months in the study. This represents an effect size of *OR* = 4.654 (*var* = 1.45, *SE* = 1.203). These employment rates were the same at the 1‐year follow‐up.

Wehman et al. (2017) tested the effects of a modified Project SEARCH program with the addition of Autism Spectrum Disorder supports, which integrated the use of applied behavior analysis. Compared to a business‐as‐usual condition (i.e., high school special education services), the modified Project SEARCH programed found after graduation, 74% of the treatment group acquired competitive, part‐time employment, compared to 6% of the comparison group (*OR* = 3.89, *var* = 1.23, *SE* = 1.11). Participants' employment status was followed‐up at the 3 months postgraduation and 12 months postgraduation. At the 3‐month follow‐up, 90% of treatment participants were employed compared to 6% of the control group (*OR* = 5.07, *var* = 1.43, *SE* = 1.19). At the 12‐month follow‐up, 87% of the treatment group and 12% of the control group were employed (*OR* = 3.99, *var* = .85, *SE* = 0.92).

Wehman et al. (2017) also measured wages earned and hours worked. However, given that the majority of the control group was not employed, it was difficult to make meaningful comparisons between the treatment and control group.

The third included study was conducted by Smith et al. (2015), who tested the effects of a virtual reality job interviewing training (VR‐JIT). At a six‐month follow‐up time point, the treatment group had 53.3% accepted a job position versus 25% of the control group (*OR* = 3.43, *var* = 0.94, *SE* = 0.97).

Together, these three included studies provide promising evidence for the effectiveness of programs similar to Project SEARCH and virtual reality job interviewing to enhance employment for individuals with ASD.

## 5 DISCUSSION

### 5.1 Summary of main results

The systematic review update identified three studies that evaluated employment outcomes for interventions for individuals with ASD. Given that prior reviews (Westbrook et al., 2012a, 2013) did not identify interventions with actual employment outcomes, the more recent emergence of evaluations of such programs is encouraging. This suggests that there is a growing body of evidence regarding interventions to enhance the employment outcomes for individuals with ASD.

All three studies identified in the review suggest that vocation‐focused programs may have positive impacts on the employment outcomes for individuals with ASD. Wehman et al. (2014) indicated that participants in Project SEARCH had higher employment rates than control participants at both 9‐month and 1‐year follow‐up time points. Adding Autism Spectrum Disorder supports, Project SEARCH in Wehman et al.'s (2017) study also demonstrated higher employment rates for treatment participants than control participants at postgraduation, 3‐month follow‐up, and 12‐month follow‐up. Smith et al. (2015) found that virtual reality job interview training was able to enhance the acceptance of job offers for treatment participants compared to control participants as well.

### 5.2 Overall completeness and applicability of evidence

Despite the small number of studies included in our review, the rigorous search process confirms a relatively complete set of available studies on interventions' effects on employment for individuals with ASD. Our update of prior systematic reviews highlights that this area is still a growing field particularly when measuring employment outcomes as a result of vocational rehabilitation with individuals with ASD.

Because of the few studies in our review, and the relatively small sample sizes, we caution readers to not overly generalize the findings from our review.

### 5.3 Quality of the evidence

In evaluating the overall quality of the evidence collected by the systematic review to support the use of specific interventions to promote the employment of individuals with autism, it should be noted that given the varying scope of the three studies as well as the heterogeneity of the population, claims of efficacy should be interpreted with caution. Additionally, although all three studies employed a RCT design, several risks of bias were identified for each. Notably, performance and detection bias seem very likely for all three studies given that none used researcher or assessor blinding in their designs. Selection bias is likely for Smith et al. (2015) since randomization and group assignment procedures were not discussed. However, threats to selection bias were addressed in Wehman et al. (2014) by the use of specific randomization through a random number generator. It is uncertain whether attrition bias was a threat to the evidence of the studies. Participant attrition was discussed in Wehman et al. (2014, 2017) as participants assigned to the control group—but not the treatment group—left the study; however, in Wehman et al. (2014), participants exited before data collection, so the risk of bias is unclear. In Wehman et al. (2017), five participants from the control group reapplied for treatment in the following group, of which four were randomly selected in treatment. Risk of reporting bias is also unlikely for Wehman et al. (2014, 2017) given the complete reporting of data in variables of interest and other confounders such as use of psychotropic medication. Missing data was not mentioned in Smith et al. (2015), thus it is uncertain the extent to which reporting bias represents a threat to validity. In addition, all studies had fairly small sample sizes: Wehman et al. (2014; *n* = 40), Smith (et al., 2015; *n* = 23), Wehman et al. (2017; *n* = 45). In both Wehman et al. (2014, 2017), authors note potential threats related to the fact that participants were limited to two (2014) and one (2017) hospital sites in the same region with largely the same staff in each study. Finally, Smith et al. (2015) notes that limited participant covariates contributed to a large amount of unexplained variance in the data.

### 5.4 Limitations and potential biases in the review process

There were several limitations in the review process. One restriction in our inclusion criteria was an employment outcome. Although there are many studies that evaluate the effectiveness of programs measuring more proximal outcomes such as interviewing skills, social interactions, or job‐related skills, there were fewer studies that actually measured employment outcomes, which was perhaps more of a distal outcome in these studies. We tried to eliminate potential biases in the review process by identifying any conflicts of interest as none of the author team was responsible for designing or evaluating any of the included interventions in the review.

### 5.5 Agreements and disagreements with other studies or reviews

#### 5.5.1 Agreement and disagreement with other reviews

The results of this study generally align with other studies and reviews with similar search criteria and methods. Most notably, many systematic reviews examining the employment of individuals with autism found limited studies meeting inclusion criteria (e.g., Di Rezze et al., 2018; Jacob et al., 2015). Generally, these similar reviews align with the results of this review both in terms of the general substance of findings as well as some of the included studies. Most recently, Di Rezze et al. (2018), in a review of employment outcomes for adults with neurodevelopmental disabilities—including but not limited to autism—also identified Wehman et al. (2014) but not Smith (2014). Given the date of initial acceptance, it is likely that Wehman et al. (2017) was not published at the time of retrieval of sampled studies. Mazzotti et al. (2016) conducted a systematic review of correlational research into outcome predictors for transition‐age youth using data from the National Longitudinal Transition Study‐2. Similarly, Haber et al. (2016) performed a meta‐analytic review of in‐school predictors of employment for students with disabilities (not autism‐specific). Although these reviews were not focused exclusively on individuals with autism and specifically examined in‐school factors, both Mazzotti et al. (2016) and Haber et al. (2016) identified work experience as a predictor of post‐school success, which aligns closely with findings from Wehman et al. (2014, 2017). Furthermore, in a more recently published study, Wehman et al. (2019) has conducted a replication study with a sample of 156 transition‐age youth with ASD and confirmed positive effects of the Project SEARCH plus ASD Supports model on employment outcomes after graduation. Although this study was published after the sampling of studies included in this review, its findings directly build on the evidence presented by Wehman et al. (2014, 2017).

#### 5.5.2 Agreement and disagreement with other studies

Although many similar studies to those included in the review were excluded due to lack of research rigor, sample, variables of interest, or other features of the inclusion criteria, the findings of this review do align with previous studies showing promise for the use of Project SEARCH and virtual reality in promoting employment‐related outcomes and skills. In particular, several studies have examined the effects of the Project SEARCH model in addressing employment gaps for individuals with autism and other intellectual and developmental disabilities. Christensen and Richardson (2017) evaluated a statewide implementation of Project SEARCH in New York state, which led to 63% of participants earning competitive employment. In a similar multisite implementation in the United Kingdom, Kaehne (2016) reported that roughly half of the participants achieved employment subsequently. However, it should be noted that the majority of research on Project SEARCH did not include specific ASD supports used by Wehman et al. (2014, 2017). Specifically, only one descriptive article (Wehman et al., 2013) and one retroactive record review (Schall et al., 2015) specified additional supports for individuals with ASD beyond the traditional Project SEARCH model.

No other studies except for Smith et al. (2015) specifically examined the effect of virtual reality job interview training (VR‐JIT) on employment outcomes for individuals with autism, though Smith et al. (2015) did build on previous findings from a feasibility study by Smith, Ginger, Wright, Wright, Taylor, et al. (2014). Other studies have examined the use of VR‐JIT with other populations including individuals with posttraumatic stress disorder (Smith, Boteler Humm, et al., 2015), severe mental illness (Smith, Fleming, Wright, Jordan, et al., 2015), psychiatric disabilities (Smith, Ginger, Wright, Wright, Boteler Humm, et al., 2014), schizophrenia (Smith, Fleming, Wright, Roberts, et al., 2015), and substance abuse disorders (Smith et al., 2016). Additionally, virtual reality has been used in other studies for individuals with autism to teach skills related to employment and adult independence such as community navigation (McMahon et al., 2015), driving safety (Bishop et al., 2017), and social competence (Stichter et al., 2014).

## 6 AUTHORS' CONCLUSIONS

### 6.1 Implications for practice and policy

#### 6.1.1 Practice

The results of this systematic review, although limited in the number of studies identified meeting inclusion criteria, continue to offer promise in improving persistently poor employment outcomes for individuals with autism through targeted intervention. Although the three studies are limited in scope, they do add continued evidence that in spite of low rates of employment nationwide for this group, pathways to employment do exist given the right programming and support. The findings of these studies largely confirm and extend previous research into the employment of individuals with autism and other developmental disabilities. For example, Wehman et al. (2014, 2017) contributed further evidence to previous research showing that the Project SEARCH internship model increased participants' likelihood of securing long‐term employment (e.g., Christensen & Richardson, 2017; Kaehne, 2016). These findings also support previous correlational studies showing that individualized work experiences predict better outcomes in competitive, integrated employment (Haber et al., 2016). Additionally, while Smith, Fleming, Wright, Losh, et al. (2015) measured a novel approach to teaching a specific career readiness skill in using virtual reality to promote interview skills, this study also contributes to a more general body of research supporting the use of technology to teach skills and promote independence in a variety of employment (Gentry et al., 2015) and school settings (Odom et al., 2015).

#### 6.1.2 Policy

In addition to their significance for practice, these studies also carry important policy implications that should be noted. First and foremost, while these three studies offer optimism in improving outcomes, the relative paucity of empirical studies meeting inclusion criteria for this review points to an urgent need for greater funding for high‐quality research and technical assistance to support the employment of individuals with autism. It should be noted that to address this lack of research in the employment of youth with disabilities, several U.S. federal agencies, led by the Social Security Administration have launched two large scale demonstration projects: the Promoting the Readiness of Minors in Supplemental Security Income (PROMISE) Initiative, and Youth Transition Demonstration (YTD) Projects (Fraker et al., 2016; Ipsen et al., 2019). Although early studies published based on PROMISE and YTD did not meet inclusion criteria for this review, the scale of national effort to investigate this issue is noteworthy. Finally, given the focus of the studies included in this review on work‐based learning (Wehman et al., 2014, 2017) and career readiness skills (Smith, Fleming, Wright, Losh, et al., 2015), it should also be mentioned that these findings do align with recent policy changes brought about by the Workforce Innovation and Opportunity Act (WIOA) of 2014 that require state vocational rehabilitation agencies to use 15% of their annual budgets on promoting these two areas of pre‐employment transition services to youth with disabilities, in addition to job exploration counseling, instruction in self‐advocacy, and counseling for opportunities in training and postsecondary education (WIOA, 2014).

We would also like to note that because our set of included studies were U.S.‐based, that differences across settings and countries may exist with regard to practice and policy implications.

### 6.2 Implications for research

Several limitations of the reviewed studies related to gaps in the existing literature and risks of bias from infrequent and inconsistent reporting should be noted. Furthermore, given the limited number of studies and interventions included, comparison of effects across studies is not possible, nor is examining how other factors related to setting, sample, staff training, and methodology related to findings. However, it is clear from the limited number of included studies that more research is needed in many areas of employment supports for people with ASD. Although a large number of publications were collected for consideration, the low number included in the final sample indicates that there is a specific need for high‐quality studies using rigorous methodologies. More closely examining the findings of the included studies, there is also a need for research to extend the current evidence in these areas. Given the intensity of intervention hours provided by the Project SEARCH Plus ASD Supports model used in Wehman (2014, 2017), future research should examine the interaction between intervention dosage and outcome, specifically whether the 900 h of business‐integrated intervention is required to achieve effects. Likewise, given the reliance on behavioral coaching techniques within the PS + ASD framework, more research is needed to examine the role of staff training as a moderating variable.

**Figure 1:**
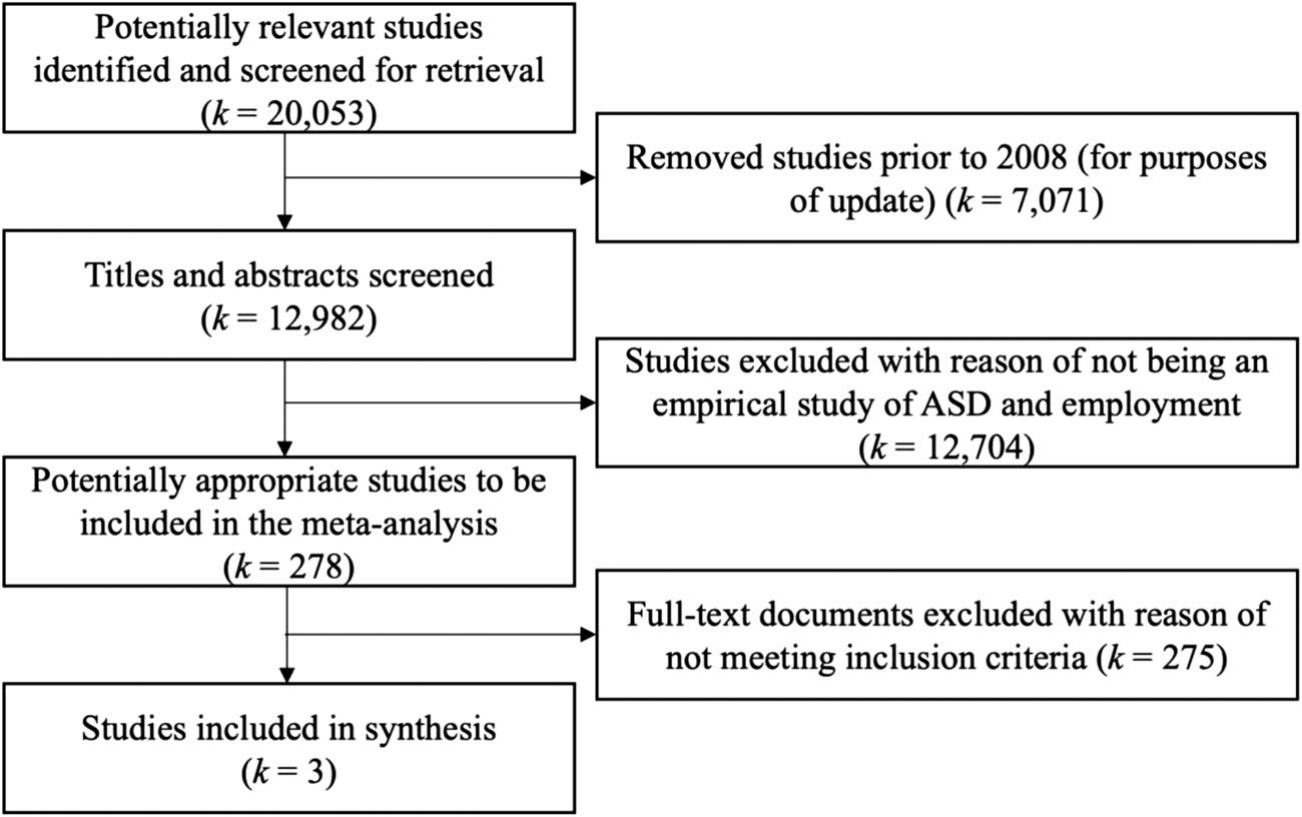
PRISMA Diagram Additional references

## PLAIN LANGUAGE SUMMARY

### Few studies but promising results on employment outcomes for individuals with ASD

Few studies evaluate interventions to improve employment outcomes for individuals with ASD. The available studies—which all have small sample sizes—suggest large effects.

### What is this review about?

The incidence of ASD is on the rise, yet individuals with ASD are gainfully employed at disproportionately lower rates than individuals without a disability. Employment interventions are vocational programs that provide training associated with access and maintenance of employment such as interviewing or vocational/social skills training.

This review looked at whether employment interventions and business‐as‐usual have different effects on the rates of employment.

**Table:** 

**What is the aim of this review?**
This Campbell systematic review update examines the effects of employment interventions for people with autism spectrum disorders. The review summarizes evidence from three randomized controlled trials.

### What studies are included?

This review uncovered three randomized studies from the USA that evaluate the effects of employment interventions for individuals with ASD. The studies spanned the period from 2014 to 2016.

### Do interventions improve employment rates for individuals with ASD?

Yes. There is an overall improvement in employment rates for individuals with ASD that participate in vocational‐related interventions.

### What do the findings of this review mean?

The main policy‐relevant findings include further consideration for how vocational rehabilitation is conducted among individuals with developmental disabilities such as ASD. Moreover, the relative paucity of empirical studies meeting inclusion criteria for this review points to an urgent need for greater funding for high‐quality research and technical assistance to support the employment of individuals with ASD.

### How up‐to‐date is this review?

The review authors searched for studies up to 2017.

## INFORMATION ABOUT THIS REVIEW

### Roles and responsibilities

 

- *Content*: Carlton Fong, Josh Taylor, and Kathleen M. Murphy.
- *Systematic review methods*: Carlton Fong.
- *Statistical analysis*: Carlton Fong.
- *Information retrieval*: Elizabeth Scalia, Amanda M. McClelland, and Aynura Berdyyeva.

Carlton J. Fong was the lead author with methodological and statistical expertise in research synthesis. He has copublished three Campbell Systematic Reviews and serves as the Editor for the Disability Coordinating Group. Josh Taylor and Kathleen M. Murphy possess expertise in disability and vocational rehabilitation. Josh Taylor, Amanda M. McClelland, and Aynura Berdyyeva have experience with systematic review screening and data extraction. Elizabeth Scalia is a librarian with expertise in information retrieval.

### SOURCES OF SUPPORT

The contents of this review were developed under grant number 90DP0077 from the National Institute on Disability, Independent Living, and Rehabilitation Research (NIDILRR). NIDILRR is a Center within the Administration for Community Living (ACL), Department of Health and Human Services (HHS). The contents of this website do not necessarily represent the policy of NIDILRR, ACL, HHS, and you should not assume endorsement by the Federal Government.

### DECLARATIONS OF INTEREST

We have no conflicts of interest to declare.

### DIFFERENCES BETWEEN PROTOCOL AND REVIEW

The current review updated two prior systematic reviews that focused on employment‐related interventions for individuals with ASD. One of the prior reviews (Westbrook et al., 2012a) and protocols (Westbrook et al., 2010) focused on adults, and the other review (Westbrook et al., 2013) and protocol (Westbrook et al., 2012b) on transition‐age youth. Thus, the updated review combined both protocols, deviating from the age restriction, to produce the current review.

### PLANS FOR UPDATING THE REVIEW

We plan to update the review in 5 years in 2026. Carlton Fong will be responsible for updating the review.

## Supporting information

Supporting information.
