# Corrigendum

**DOI:** 10.1002/cl2.1193

**Published:** 2021-09-14

The authors of Fong et al. (2021) have supplied the following correction to their article. In the Methods section under “3.2.1 Electronic searches” there should be an indication that the full search strategies can be found in an extra supplementary document.

In “4.1.6 Participants,” under the subsection Interventions, the authors had mistakenly reported that participants in the Smith et al. (2015) study accessed the job interview application using wearable virtual reality headsets. They wanted to correct this statement and clarify that headsets were not used in their study.

## Supporting information

Supporting Information
