# Supplementary material for: Interventions for improving employment outcomes for persons with autism spectrum disorders: A systematic review update
Source: Campbell Syst Rev. 2021 Jul 3;17(3):e1185. doi: 10.1002/cl2.1185 (PMC8354554; doi:10.1002/cl2.1185)
Supplement: Supplementary file 1 — Supporting information. [file CL2-17-e1185-s001.doc]

# Coding Form:

# Employment for Individuals with Autism Studies

**Full Citation (APA style):**

**Is this an Intervention Study?**

**Yes**

**Unclear**

**No, then STOP!**

**Were the Participants diagnosed with ASD?**

**Yes**

**Unclear**

**No, then STOP!**

1. **Publication Source:**

Journal Article

Conference paper

Master/Doctoral Thesis

Technical Report

Organizational Report

Book or Book Chapter

Other:

1. **Subject Characteristics (pg.      )**

|  | Groups | Pretest (n) | Posttest (n) | Attrition (n) | 1^st^ Followup (n) | F Attrition (n) | Age (yr; mos) | %Male |
| --- | --- | --- | --- | --- | --- | --- | --- | --- |
| T: |  |  |  |  |  |  |  |  |
|  |  |  |  |  |  |  |  |  |
| Co: |  |  |  |  |  |  |  |  |
|  |  |  |  |  |  |  |  |  |

Note: For groups, T=treatment, Co=comparison; “Attrition” is the difference between the pretest and posttest “n” and “F_Attrition” is the difference between the posttest and 1^st^ followup “n.”

Comments:

**III. Sample Source (pg.      )**

Public Agency

Private Agency

Not Reported

Other

Comments:

**IV. SES (pg.      )**

Low

Low-Middle

Middle

Middle-Upper

Upper

Labeled Mixed

Unlabeled Mixed

Unclear

Not Reported

Comments:

**V. Education (pg.      )**

Some High School

High School Graduate

Some College

College Diploma

Other:

Comments:

**VI. Study Community Setting (pg.      )**

Urban  Suburban  Rural  NR

Comments:       Geographic Setting:

**VII. Employment Setting (pg.      )**

Integrated/Competitive (work is performed alongside non-disabled co-workers)

Non-integrated (work is performed entirely alongside disabled co-workers)

Supported Employment

Not Reported

Comments:

**VIII. Participant Classification (pg.      )**

ASD

Autism

Asperger’s (HFA)

PDD-NOS (Pervasive)

Rhett Syndrome

Childhood Disintegrative Disorder

Comments:

# IX. Classification Severity Level (pg.      )

Mild  Moderate-Severe

Mild-Moderate  Severe

Moderate  Mixed

Not Reported

Comments:

**X. Race/Ethnicity (pg.      )**

**T C**

African-American **%      %**

American-Indian **%      %**

Asian **%      %**

Hispanic/Latino **%      %**

White/Caucasian **%      %**

Mixed **%      %**

Other:       **%      %**

Not Reported

Comments:

# Intervention Characteristics (pg.      )

**XI. Describe Intervention Characteristics (pg.      )**

Details of Intervention intended for treatment/comparison groups including how and when administered. What were the components of the intervention? Who was involved?

Average Length of Intervention Program **(pg.      ):**

Length of time of participation activity – frequency **(pg.      ):**       per

Number of Sessions **(pg.      ):**

**Primary Type of Employment:**

Wholesale Trade

Retail Trade

Transportation & Warehousing

Information

Finance

Professional

Education & health

Leisure & Hospitality

Other Service:

Goods-processing Industries

Construction

Manufacturing

Other Service:

Public Administration

Local government

State government

Federal government  Other Service:

Comments:

**XII. Outcome Measure(s):**

1. Length of Time to Place in Employment:
2. Length of Time Employed:
3. Re-employments Included:  Yes  No  Not Reported
4. **Employment Status:**  **Full Time**  **Part Time**
5. Hours worked per week:
6. Post-placement Hourly Wages:
7. Dropped Out Before Placement Occurred:
8. Employer Evaluation:
9. Co-Worker Evaluation:
10. Participant Evaluation:

Comments:

**Design Characteristics (pg**      **)**

**XIII. Research Design Characteristics:**

Which of the following research design types were used to examine the impact of program effects for employment placement?

RCT Individual Randomized Design

RCT Group Randomized Design

Quasi-Experiment: Equivalent Comparison Design (individuals)

Quasi-Experiment: Equivalent Comparison Design (groups)

Quasi-Experiment: Nonequivalent Comparison Design (individuals)

Quasi-Experiment: Nonequivalent Comparison Design (groups)

Other Design:

If none of the above then **STOP!**

Comments:

**XIV. Method of Random Assignment (pg**      **)**

Random Number Generation

Coin Flip

Envelope

Other

NR

Comments:

**XV. Recruitment Pool (pg**      **)**

Referral  Criterion       Pre-placement Test Score

Existing Group  Volunteer Waiting List

Other        NR

Comments:

**XVI. Blinding**

Researcher **(pg**      **)**

Participant **(pg**      **)**

Intervener **(pg**      **)**

Assessor **(pg**      **)**

Employer **(pg**      **)**

Other       **(pg**      **)**

Comments:

**XVII. Fidelity of Implementation**

Intervention implemented as described **(pg**      **)**   No  Yes  NR

Comments:

**XVIII Effect Size Characteristics (Use d-Index Value if Provided)**

Groups Compared: **Group 1:**       **Group 2:**

Outcomes

Groups **1 2 1 2 1 2 1 2**

Mean ____ ____ ____ ____ ____ ____ ____ ____

SD ____ ____ ____ ____ ____ ____ ____ ____

*d*-index ____ ____ ____ ____ ____ ____ ____ ____

F value ____ ____ ____ ____ ____ ____ ____ ____

p value ____ ____ ____ ____ ____ ____ ____ ____

t value ____ ____ ____ ____ ____ ____ ____ ____

Odds ratio ____ ____ ____ ____ ____ ____ ____ ____

Chi-square ____ ____ ____ ____ ____ ____ ____ ____

N-Employed ____ ____ ____ ____ ____ ____ ____ ____

N-Unemployed ____ ____ ____ ____ ____ ____ ____ ____
