# Supplementary material for: Corrigendum
Source: Campbell Syst Rev. 2021 Sep 14;17(3):e1193. doi: 10.1002/cl2.1193 (PMC8988691; doi:10.1002/cl2.1193)
Supplement: Supplementary file 1 — Supporting Information [file CL2-17-e1193-s001.docx]

Search Strategies

**Search in PubMed on 06/05/2017:**

(Autism Spectrum Disorder[mh] OR Autistic Disorder[mh] OR Asperger Syndrome[mh] OR "Child Development Disorders, Pervasive"[mh] OR Rett Syndrome[mh] OR Autis* OR Asperger* OR ASD[tiab] OR Pervasive Development Disorder* OR Pervasive developmental disorder* OR Pervasive Child Development Disorder* OR Pervasive Child Developmental Disorder* OR PDD-NOS OR Rett syndrome* OR Rett's syndrome* OR "Rett disorder" OR "Rett's disorder" OR Childhood Disintegrative Disorder* OR "Heller's syndrome" OR **"**Heller syndrome")

AND

(Employment[mh] OR "Employment, Supported"[mh] OR "Rehabilitation, Vocational"[mh] OR Vocational Guidance[mh] OR Work[mh] OR Work Performance[mh] OR Workplace[mh] OR Occupations[mh] OR Career Choice[mh] OR Career Mobility[mh] OR "Return to Work"[mh] OR Occupational medicine[mh] OR Occupational health[mh] OR Occupational health services[mh] OR Occupational Therapy[mh] OR Job Application[mh] OR Employ* OR pre-employ* OR Work OR Working OR workplace* OR worker* OR worksite* OR workab* OR Vocation* OR occupation* OR trade* OR career* OR job*)

Filters activated: Publication date from 2008/01/01

3217 results

Then used same search strings in PubMed as above but used two different date codes.

(Autism Spectrum Disorder[mh] OR Autistic Disorder[mh] OR Asperger Syndrome[mh] OR "Child Development Disorders, Pervasive"[mh] OR Rett Syndrome[mh] OR Autis* OR Asperger* OR ASD[tiab] OR Pervasive Development Disorder* OR Pervasive developmental disorder* OR Pervasive Child Development Disorder* OR Pervasive Child Developmental Disorder* OR PDD-NOS OR Rett syndrome* OR Rett's syndrome* OR "Rett disorder" OR "Rett's disorder" OR Childhood Disintegrative Disorder* OR "Heller's syndrome" OR **"**Heller syndrome")

AND

(Employment[mh] OR "Employment, Supported"[mh] OR "Rehabilitation, Vocational"[mh] OR Vocational Guidance[mh] OR Work[mh] OR Work Performance[mh] OR Workplace[mh] OR Occupations[mh] OR Career Choice[mh] OR Career Mobility[mh] OR "Return to Work"[mh] OR Occupational medicine[mh] OR Occupational health[mh] OR Occupational health services[mh] OR Occupational Therapy[mh] OR Job Application[mh] OR Employ* OR pre-employ* OR Work OR Working OR workplace* OR worker* OR worksite* OR workab* OR Vocation* OR occupation* OR trade* OR career* OR job*)

AND

2008/01/01:2017/06/05[mhda]

3272 results

(Autism Spectrum Disorder[mh] OR Autistic Disorder[mh] OR Asperger Syndrome[mh] OR "Child Development Disorders, Pervasive"[mh] OR Rett Syndrome[mh] OR Autis* OR Asperger* OR ASD[tiab] OR Pervasive Development Disorder* OR Pervasive developmental disorder* OR Pervasive Child Development Disorder* OR Pervasive Child Developmental Disorder* OR PDD-NOS OR Rett syndrome* OR Rett's syndrome* OR "Rett disorder" OR "Rett's disorder" OR Childhood Disintegrative Disorder* OR "Heller's syndrome" OR **"**Heller syndrome")

AND

(Employment[mh] OR "Employment, Supported"[mh] OR "Rehabilitation, Vocational"[mh] OR Vocational Guidance[mh] OR Work[mh] OR Work Performance[mh] OR Workplace[mh] OR Occupations[mh] OR Career Choice[mh] OR Career Mobility[mh] OR "Return to Work"[mh] OR Occupational medicine[mh] OR Occupational health[mh] OR Occupational health services[mh] OR Occupational Therapy[mh] OR Job Application[mh] OR Employ* OR pre-employ* OR Work OR Working OR workplace* OR worker* OR worksite* OR workab* OR Vocation* OR occupation* OR trade* OR career* OR job*)

AND

2008/01/01:2017/06/05[crdt]

3226 results

And then OR the 3 sets together.

(#21 OR #20 OR #19)

3292 results, found a few duplicates, left with **3289 unique results.**

**Search in PsycINFO on EBSCO interface on 06/22/2017:**

(DE "Autism Spectrum Disorders" OR DE "Autistic Thinking" OR DE "Rett Syndrome" OR Autis* OR Asperger* OR ASD OR Pervasive Development Disorder* OR Pervasive developmental disorder* OR Pervasive Child Development Disorder* OR Pervasive Child Developmental Disorder* OR PDD-NOS OR Rett syndrome* OR Rett's syndrome* OR "Rett disorder" OR "Rett's disorder" OR Childhood Disintegrative Disorder* OR "Heller's syndrome" OR **"**Heller syndrome") in Select a field, optional

AND

(DE "Employment Status" OR DE "Self-Employment" OR DE "Supported Employment" OR DE "Work Adjustment Training" OR DE "Vocational Rehabilitation" OR DE "Vocational Evaluation" OR DE "Vocational Education" OR DE "Cooperative Education" OR DE "Disability Management" OR DE "Career Education" OR DE "Occupations" OR DE "Nontraditional Careers" OR DE "Employability" OR DE "Reemployment" OR DE "School to Work Transition" OR DE "Transition Planning" OR DE "Job Involvement" OR DE "Occupations" OR DE "Occupational Adjustment" OR DE "Occupational Guidance" OR DE "Occupational Choice" OR DE "Occupational Success" OR DE "Occupational Success Prediction" OR DE "Occupational Preference" OR DE "Occupational Aspirations" OR DE "Occupational Interests" OR DE "Occupational Interest Measures" OR DE "Kuder Occupational Interest Survey" OR DE "Strong Vocational Interest Blank" OR DE "Occupational Health" OR DE "Occupational Therapy" OR Employ* OR pre-employ* OR Work OR Working OR workplace* OR worker* OR worksite* OR workab* OR Vocation* OR occupation* OR trade* OR career* OR job*) in Select a field, optional

Limiters: Publication Year: 2008-

7269 results

(DE "Autism Spectrum Disorders" OR DE "Autistic Thinking" OR DE "Rett Syndrome" OR Autis* OR Asperger* OR ASD OR Pervasive Development Disorder* OR Pervasive developmental disorder* OR Pervasive Child Development Disorder* OR Pervasive Child Developmental Disorder* OR PDD-NOS OR Rett syndrome* OR Rett's syndrome* OR "Rett disorder" OR "Rett's disorder" OR Childhood Disintegrative Disorder* OR "Heller's syndrome" OR **"**Heller syndrome") in Select a field, optional

AND

(DE "Employment Status" OR DE "Self-Employment" OR DE "Supported Employment" OR DE "Work Adjustment Training" OR DE "Vocational Rehabilitation" OR DE "Vocational Evaluation" OR DE "Vocational Education" OR DE "Cooperative Education" OR DE "Disability Management" OR DE "Career Education" OR DE "Occupations" OR DE "Nontraditional Careers" OR DE "Employability" OR DE "Reemployment" OR DE "School to Work Transition" OR DE "Transition Planning" OR DE "Job Involvement" OR DE "Occupations" OR DE "Occupational Adjustment" OR DE "Occupational Guidance" OR DE "Occupational Choice" OR DE "Occupational Success" OR DE "Occupational Success Prediction" OR DE "Occupational Preference" OR DE "Occupational Aspirations" OR DE "Occupational Interests" OR DE "Occupational Interest Measures" OR DE "Kuder Occupational Interest Survey" OR DE "Strong Vocational Interest Blank" OR DE "Occupational Health" OR DE "Occupational Therapy" OR Employ* OR pre-employ* OR Work OR Working OR workplace* OR worker* OR worksite* OR workab* OR Vocation* OR occupation* OR trade* OR career* OR job*) in Select a field, optional

AND

20080101- in RD Release Date

7650 results

S5 OR S4

7651 results, file emailed in RIS format, imported into EndNote, de-duplicated, left with **6089 unique results.**

**Search in Psychology and Behavioral Sciences Collection on EBSCO interface on 06/26/2017:**

(DE "AUTISM" OR DE "ASPERGER'S syndrome" OR DE "AUTISM in adolescence" OR DE "AUTISM in adults" OR DE "AUTISM in children" OR DE "ASPERGER'S syndrome in adults" OR DE "AUTISM spectrum disorders" OR DE "AUTISM spectrum disorders in women" OR DE "PATHOLOGICAL demand avoidance syndrome" OR DE "PERVASIVE developmental disorder not otherwise specified" OR DE "RETT syndrome" OR DE "PERVASIVE child development disorders" OR DE "CHILDHOOD disintegrative disorder" OR Autis* OR Asperger* OR ASD OR Pervasive Development Disorder* OR Pervasive developmental disorder* OR Pervasive Child Development Disorder* OR Pervasive Child Developmental Disorder* OR PDD-NOS OR Rett syndrome* OR Rett's syndrome* OR "Rett disorder" OR "Rett's disorder" OR Childhood Disintegrative Disorder* OR "Heller's syndrome" OR **"**Heller syndrome") in Select a field, optional

AND

(DE "EMPLOYMENT (Economic theory)" OR DE "EMPLOYMENT of people with disabilities" OR DE "PEOPLE with disabilities -- Vocational guidance" OR DE "SUPPORTED employment" OR DE "EMPLOYMENT reentry" OR DE "VOCATIONAL guidance" OR DE "WORK" OR DE "COUNSELING in vocational education" OR DE "SCHOOL-to-work transition" OR DE "CAREER education" OR DE "OCCUPATIONAL training" OR DE "VOCATIONAL education" OR DE "EMPLOYEE training" OR DE "APPRENTICESHIP programs" OR DE "COOPERATIVE education" OR DE "INTERNSHIP programs" OR DE "TECH Prep programs" OR DE "VOCATIONAL high schools" OR DE "TECHNICAL education" OR DE "VOCATIONAL schools" OR DE "TECHNICAL institutes" OR DE "VOCATIONAL rehabilitation" OR DE "EMPLOYABILITY" OR DE "VOCATIONAL evaluation" OR DE "OCCUPATIONS" OR DE "OCCUPATIONAL medicine" OR DE "OCCUPATIONAL health services" OR Employ* OR pre-employ* OR Work OR Working OR workplace* OR worker* OR worksite* OR workab* OR Vocation* OR occupation* OR trade* OR career* OR job*) in Select a field, optional

Limiters: 20080101-

1145 results, file emailed in RIS format, imported into EndNote, de-duplicated, left with **447 unique results.**

**Search in ERIC on EBSCO interface on 06/28/2017:**

(DE "Autism" OR DE "Pervasive Developmental Disorders" OR DE "Asperger Syndrome" OR Autis* OR Asperger* OR ASD OR Pervasive Development Disorder* OR Pervasive developmental disorder* OR Pervasive Child Development Disorder* OR Pervasive Child Developmental Disorder* OR PDD-NOS OR Rett syndrome* OR Rett's syndrome* OR "Rett disorder" OR "Rett's disorder" OR Childhood Disintegrative Disorder* OR "Heller's syndrome" OR **"**Heller syndrome") in Select a field, optional

AND

(DE "Employment" OR DE "Supported Employment" OR DE "Teleworking" OR DE "Youth Employment" OR DE "Self Employment" OR DE "Employment Potential" OR DE "Career Academies" OR DE "Vocational Schools" OR DE "Career Education" OR DE "Vocational Education" OR DE "Vocational High Schools" OR DE "Vocational Training Centers" OR DE "Prevocational Education" OR DE "School Business Relationship" OR DE "Business Education" OR DE "Cooperative Education" OR DE "Technical Education" OR DE "Tech Prep" OR DE "Apprenticeships" OR DE "Field Experience Programs" OR DE "Trade and Industrial Education" OR DE "Internship Programs" OR DE "Career Counseling" OR DE "Vocational Adjustment" OR DE "Vocational Evaluation" OR DE "Work Experience Programs" OR DE "Job Training" OR DE "Individualized Transition Plans" OR DE "Individualized Education Programs" OR DE "Transitional Programs" OR DE "Equal Opportunities (Jobs)" OR DE "Community Based Instruction (Disabilities)" OR DE "Post High School Guidance" OR DE "Vocational Rehabilitation" OR DE "Workplace Learning" OR DE "Work Environment" OR DE "Occupations" OR DE "Careers" OR DE "Occupational Therapy" OR Employ* OR pre-employ* OR Work OR Working OR workplace* OR worker* OR worksite* OR workab* OR Vocation* OR occupation* OR trade* OR career* OR job*) in Select a field, optional

Limiters: Date Published: 20080101-

1380 results

(DE "Autism" OR DE "Pervasive Developmental Disorders" OR DE "Asperger Syndrome" OR Autis* OR Asperger* OR ASD OR Pervasive Development Disorder* OR Pervasive developmental disorder* OR Pervasive Child Development Disorder* OR Pervasive Child Developmental Disorder* OR PDD-NOS OR Rett syndrome* OR Rett's syndrome* OR "Rett disorder" OR "Rett's disorder" OR Childhood Disintegrative Disorder* OR "Heller's syndrome" OR **"**Heller syndrome") in Select a field, optional

AND

(DE "Employment" OR DE "Supported Employment" OR DE "Teleworking" OR DE "Youth Employment" OR DE "Self Employment" OR DE "Employment Potential" OR DE "Career Academies" OR DE "Vocational Schools" OR DE "Career Education" OR DE "Vocational Education" OR DE "Vocational High Schools" OR DE "Vocational Training Centers" OR DE "Prevocational Education" OR DE "School Business Relationship" OR DE "Business Education" OR DE "Cooperative Education" OR DE "Technical Education" OR DE "Tech Prep" OR DE "Apprenticeships" OR DE "Field Experience Programs" OR DE "Trade and Industrial Education" OR DE "Internship Programs" OR DE "Career Counseling" OR DE "Vocational Adjustment" OR DE "Vocational Evaluation" OR DE "Work Experience Programs" OR DE "Job Training" OR DE "Individualized Transition Plans" OR DE "Individualized Education Programs" OR DE "Transitional Programs" OR DE "Equal Opportunities (Jobs)" OR DE "Community Based Instruction (Disabilities)" OR DE "Post High School Guidance" OR DE "Vocational Rehabilitation" OR DE "Workplace Learning" OR DE "Work Environment" OR DE "Occupations" OR DE "Careers" OR DE "Occupational Therapy" OR Employ* OR pre-employ* OR Work OR Working OR workplace* OR worker* OR worksite* OR workab* OR Vocation* OR occupation* OR trade* OR career* OR job*) in Select a field, optional

AND

2008- in EM Date Added

1472 results

S1 OR S2

1473 results, file emailed in RIS format, imported into EndNote, de-duplicated, left with **679 unique results.**

**Search in Education Source on EBSCO interface on 06/30/2017:**

(DE "Autism" OR DE "Autism spectrum disorders" OR DE "Asperger's syndrome" OR DE "Autism in adolescence" OR DE "Autism spectrum disorders in women" OR DE "Pervasive developmental disorder not otherwise specified" OR DE "Autistic people" OR DE "Education of autistic people" OR DE "Higher education of autistic people" OR Autis* OR Asperger* OR ASD OR Pervasive Development Disorder* OR Pervasive developmental disorder* OR Pervasive Child Development Disorder* OR Pervasive Child Developmental Disorder* OR PDD-NOS OR Rett syndrome* OR Rett's syndrome* OR "Rett disorder" OR "Rett's disorder" OR Childhood Disintegrative Disorder* OR "Heller's syndrome" OR **"**Heller syndrome") in Select a field, optional

AND

(DE "Employment (Economic theory)" OR DE "Supported employment" OR DE "Employment of people with disabilities" OR DE "Employment reentry" OR DE "Employability" OR DE "Vocational rehabilitation" OR DE "Educational counseling" OR DE "Vocational guidance" OR DE "Vocational evaluation" OR DE "Vocational education" OR DE "Vocational schools" OR DE "Vocational training centers" OR DE "Career education" OR DE "Career academies" OR DE "Internship programs" OR DE "Apprenticeship programs" OR DE "Education & training services industry" OR DE "Technical education" OR DE "Tech Prep programs" OR DE "Vocational high schools" OR DE "Technical institutes" OR DE "School-to-work transition" OR DE "Link courses (Education)" OR DE "College placement services" OR DE "Cooperative education" OR DE "Individualized transition plans" OR DE "Transitional programs (Education)" OR DE "Occupations" OR DE "Occupational training" OR DE "Employment of youth" OR Employ* OR pre-employ* OR Work OR Working OR workplace* OR worker* OR worksite* OR workab* OR Vocation* OR occupation* OR trade* OR career* OR job*) in Select a field, optional

Limiters: Published Date: 20080101-

2452 results

(DE "Autism" OR DE "Autism spectrum disorders" OR DE "Asperger's syndrome" OR DE "Autism in adolescence" OR DE "Autism spectrum disorders in women" OR DE "Pervasive developmental disorder not otherwise specified" OR DE "Autistic people" OR DE "Education of autistic people" OR DE "Higher education of autistic people" OR Autis* OR Asperger* OR ASD OR Pervasive Development Disorder* OR Pervasive developmental disorder* OR Pervasive Child Development Disorder* OR Pervasive Child Developmental Disorder* OR PDD-NOS OR Rett syndrome* OR Rett's syndrome* OR "Rett disorder" OR "Rett's disorder" OR Childhood Disintegrative Disorder* OR "Heller's syndrome" OR **"**Heller syndrome") in Select a field, optional

AND

(DE "Employment (Economic theory)" OR DE "Supported employment" OR DE "Employment of people with disabilities" OR DE "Employment reentry" OR DE "Employability" OR DE "Vocational rehabilitation" OR DE "Educational counseling" OR DE "Vocational guidance" OR DE "Vocational evaluation" OR DE "Vocational education" OR DE "Vocational schools" OR DE "Vocational training centers" OR DE "Career education" OR DE "Career academies" OR DE "Internship programs" OR DE "Apprenticeship programs" OR DE "Education & training services industry" OR DE "Technical education" OR DE "Tech Prep programs" OR DE "Vocational high schools" OR DE "Technical institutes" OR DE "School-to-work transition" OR DE "Link courses (Education)" OR DE "College placement services" OR DE "Cooperative education" OR DE "Individualized transition plans" OR DE "Transitional programs (Education)" OR DE "Occupations" OR DE "Occupational training" OR DE "Employment of youth" OR Employ* OR pre-employ* OR Work OR Working OR workplace* OR worker* OR worksite* OR workab* OR Vocation* OR occupation* OR trade* OR career* OR job*) in Select a field, optional

AND

ED 20080101- in Select a field, optional

3065 results

S1 OR S2

3068 results, file emailed in RIS format, imported into EndNote, de-duplicated, left with **1638 unique results.**

**Search in SocINDEX on EBSCO interface on 07/07/2017:**

(DE "AUTISM" OR DE "AUTISM in adolescence" OR DE "AUTISM in adults" OR DE "AUTISM in children" OR DE "AUTISM spectrum disorders" OR DE "AUTISM spectrum disorders in women" OR DE "AUTISTIC children" OR DE "AUTISTIC youth" OR Autis* OR Asperger* OR ASD OR Pervasive Development Disorder* OR Pervasive developmental disorder* OR Pervasive Child Development Disorder* OR Pervasive Child Developmental Disorder* OR PDD-NOS OR Rett syndrome* OR Rett's syndrome* OR "Rett disorder" OR "Rett's disorder" OR Childhood Disintegrative Disorder* OR "Heller's syndrome" OR **"**Heller syndrome") in Select a field, optional

AND

(DE "EMPLOYMENT (Economic theory)" OR DE "PEOPLE with disabilities -- Employment" OR DE "SUPPORTED employment" OR DE "SELF-employment" OR DE "EMPLOYABILITY" OR DE "VOCATIONAL rehabilitation" OR DE "WORK" OR DE "WORK environment" OR DE "OCCUPATIONS" OR DE "OCCUPATIONAL therapy" OR DE "OCCUPATIONAL health services" OR DE "OCCUPATIONAL medicine" OR DE "CAREER education" OR DE "OCCUPATIONAL training" OR DE "VOCATIONAL guidance" OR DE "EDUCATIONAL counseling" OR DE "VOCATIONAL education" OR DE "INTERNSHIP programs" OR DE "PRACTICUMS" OR DE "TECHNICAL institutes" OR Employ* OR pre-employ* OR Work OR Working OR workplace* OR worker* OR worksite* OR workab* OR Vocation* OR occupation* OR trade* OR career* OR job*) in Select a field, optional

Limiters: Date of Publication: 20080101-

414 results, imported into EndNote, de-duplicated, left with **170 unique results.**

**Search in Web of Science Core Collection on 07/12/2017:**

Note: Web of Science Core Collection includes the following databases:

Science Citation Index Expanded (SCI-EXPANDED) --1996-present

Social Sciences Citation Index (SSCI) --1996-present

Arts & Humanities Citation Index (A&HCI) --1996-present

Conference Proceedings Citation Index- Science (CPCI-S) --1996-present

Conference Proceedings Citation Index- Social Science & Humanities (CPCI-SSH) --1996-present

Book Citation Index– Science (BKCI-S) --2005-present

Book Citation Index– Social Sciences & Humanities (BKCI-SSH) --2005-present

Emerging Sources Citation Index (ESCI) --2015-present

(Autis* OR Asperger* OR ASD OR Pervasive Development Disorder* OR Pervasive developmental disorder* OR Pervasive Child Development Disorder* OR Pervasive Child Developmental Disorder* OR PDD-NOS OR Rett syndrome* OR Rett's syndrome* OR "Rett disorder" OR "Rett's disorder" OR Childhood Disintegrative Disorder* OR "Heller's syndrome" OR **"**Heller syndrome") in Topic

AND

(Employ* OR pre-employ* OR Work OR Working OR workplace* OR worker* OR worksite* OR workab* OR Vocation* OR occupation* OR trade* OR career* OR job*) in Topic

From: 2008-2017

5235 results, imported into EndNote, de-duplicated, left with **2,371 unique results**

**Search in Cochrane Library on the Wiley interface on 7/18/2017:**

Note: Cochrane Library includes the following databases:

Cochrane Database of Systematic Reviews (CDSR)

Cochrane Central Register of Controlled Trials (CENTRAL)

Cochrane Methodology Register (CMR)

Database of Abstracts of Reviews of Effects (DARE)

Health Technology Assessment Database (HTA)

NHS Economic Evaluation Database (NHS EED)

ID Search Hits

#1 MeSH descriptor: [Autism Spectrum Disorder] explode all trees 71

#2 MeSH descriptor: [Autistic Disorder] explode all trees 602

#3 MeSH descriptor: [Asperger Syndrome] explode all trees 49

#4 MeSH descriptor: [Child Development Disorders, Pervasive] explode all trees 926

#5 MeSH descriptor: [Rett Syndrome] explode all trees 21

#6 Autis* or Asperger* or ASD or Pervasive Development Disorder or Pervasive developmental disorder or Pervasive Child Development Disorder or Pervasive Child Developmental Disorder or PDD-NOS or Rett syndrome or Rett's syndrome or "Rett disorder" or "Rett's disorder" or Childhood Disintegrative Disorder or "Heller's syndrome" or "Heller syndrome":ti,ab,kw (Word variations have been searched) 2053

#7 MeSH descriptor: [Employment] explode all trees 1669

#8 MeSH descriptor: [Employment, Supported] explode all trees 107

#9 MeSH descriptor: [Rehabilitation, Vocational] explode all trees 421

#10 MeSH descriptor: [Vocational Guidance] explode all trees 34

#11 MeSH descriptor: [Work] explode all trees 487

#12 MeSH descriptor: [Work Performance] explode all trees 7

#13 MeSH descriptor: [Workplace] explode all trees 725

#14 MeSH descriptor: [Occupations] explode all trees 167

#15 MeSH descriptor: [Career Choice] explode all trees 56

#16 MeSH descriptor: [Career Mobility] explode all trees 11

#17 MeSH descriptor: [Return to Work] explode all trees 133

#18 MeSH descriptor: [Occupational Medicine] explode all trees 70

#19 MeSH descriptor: [Occupational Health] explode all trees 597

#20 MeSH descriptor: [Occupational Health Services] explode all trees 402

#21 MeSH descriptor: [Occupational Therapy] explode all trees 702

#22 MeSH descriptor: [Job Application] explode all trees 22

#23 Employ* or pre-employ* or Work or Working or workplace* or worker* or worksite* or workab* or Vocation* or occupation* or trade* or career* or job*:ti,ab,kw (Word variations have been searched) 56529

#24 (#1 or #2 or #3 or #4 or #5 or #6) 2053

#25 (#7 or #8 or #9 or #10 or #11 or #12 or #13 or #14 or #15 or #16 or #17 or #18 or #19 or #20 or #21 or #22 or #23) 56545

#26 #24 and #25 Publication Year from 2008 162

#27 #24 and #25 Online Publication Date from Jan 2008 5

#28 #26 or #27 162

Here is the breakdown of the 162 results (before de-duplication):

Cochrane Database of Systematic Reviews (CDSR) 5

Database of Abstracts of Reviews of Effect (DARE) 3

Cochrane Central Register of Controlled Trials (CENTRAL) 152

Health Technology Assessment Database (HTA) 1

NHS Economic Evaluation Database (NHS EED) 1

162 results, imported into EndNote, de-duplicated, left with **54 unique results.**

**Searches in REHABDATA at** [**http://www.naric.com/?q=en/SearchRehabdata**](http://www.naric.com/?q=en/SearchRehabdata) **on 7/26/2017:**

Search all fields in REHABDATA for records:

With all of these words: autis* employment

Year of Publication between: 2008 and 2017; Yes, include international research

140 results

Then, keeping the same search parameters, used these word combinations also:

Asperger* employment

4

Pervasive Developmental Disorder employment

2

(Tested the same terms with "pre-employment" and any resulting citations are already captured in the "employment" results sets)

autis* workplace

15

Asperger* workplace

0

Pervasive Developmental Disorder workplace

0

autis* vocational

126

Asperger* vocational

5

Pervasive Developmental Disorder vocational

3

295 total results, imported into EndNote, de-duplicated, left with **78 unique results**

**Search in WorldCat at** [**http://www.worldcat.org/advancedsearch**](http://www.worldcat.org/advancedsearch) **on 7/21/2017:**

(Autis* OR Asperger* OR ASD OR "Pervasive Development Disorder*" OR "Pervasive developmental disorder*" OR "Pervasive Child Development Disorder*" OR "Pervasive Child Developmental Disorder*" OR PDD-NOS OR "Rett syndrome*" OR "Rett's syndrome*" OR "Rett disorder" OR "Rett's disorder" OR "Childhood Disintegrative Disorder*" OR "Heller's syndrome" OR **"**Heller syndrome") in Keyword

AND

(Employ* OR "pre-employ*" OR Work OR Working OR workplace* OR worker* OR worksite* OR workab* OR Vocation* OR occupation* OR trade* OR career* OR job*) in Keyword

Year: 2008 to 2018

Format: Book (this includes dissertations/theses)

150 results, imported into EndNote, de-duplicated, left with **133 unique results.**

**Browsed EPPI-Centre's systematic reviews on 7/21/2017:**

Browsed the index of systematic reviews categorized by topic at this page <https://eppi.ioe.ac.uk/cms/Default.aspx?tabid=60>

**1 result**

**Browsed AHRQ's Effective Health Care Program reports on 7/26/2017:**

Performed a simple search at <https://www.effectivehealthcare.ahrq.gov/index.cfm/search-for-guides-reviews-and-reports/>

Health Condition drop-down menu: selected "All Developmental delays, ADHD, Autism"

Keyword box: employment

Only 1 looked relevant, but is a duplicate, already pulled by PubMed search.

0 results

**Browsed Joanna Briggs Institute's systematic reviews on 7/26/2017:**

JBI Database of Systematic Reviews and Implementation Reports at <http://journals.lww.com/jbisrir/Pages/default.aspx> .

Abstract: autis*

AND

Abstract: employment

Only 1 result, but is not relevant.

0 results

**Browsed Social Care Institute for Excellence's resources and services page and their NCCSC page on 7/27/2017:**

Browsed these two pages: <http://www.scie.org.uk/atoz/> and <http://www.scie.org.uk/nccsc/index.asp>

**4 results**

**Searches in Social Care Online at** [**http://www.scie-socialcareonline.org.uk**](http://www.scie-socialcareonline.org.uk) **on 7/28/2017:**

All fields exact: autism

AND

All fields exact: employment

Publication year: 2008-2017

71 results

Keeping same search criteria, used these terms also:

Asperger AND employment

7

Aspergers AND employment

19

"Pervasive Developmental Disorder" AND employment (used other phrase variations as well)

0

Tried all terms with "Pre-employment" but 0 results

autism AND vocational

5

Asperger AND vocational

0

Aspergers AND vocational

0

"Pervasive Developmental Disorder" AND vocational

0

102 results, imported into EndNote, de-duplicated, left with **57 unique results.**

**Searches in LearnTechLib at** [**https://www.learntechlib.org/search/advanced/**](https://www.learntechlib.org/search/advanced/) **on 8/1/2017:**

Simple Search, Keyword search

With all of the words: autism employment

In the Limits area:

All records

Year Published 2008 to 2018

All Publications

47 results

Then, keeping the same search parameters, tried these word combinations also:

autistic employment 12 results

Asperger employment 7 results

Asperger's employment 3 results

Pervasive Developmental Disorder employment 3 results

Autism "pre-employment" 0 results

Autistic "pre-employment" 0 results

Asperger "pre-employment" 0 results

Asperger's "pre-employment" 0 results

Pervasive Developmental Disorder "pre-employment" 0 results

autism workplace 18 results

autistic workplace 7 results

Asperger workplace 2 results

Asperger's workplace 1 result

Pervasive Developmental Disorder workplace 1 result

autism vocational 89 results

autistic vocational 10 results

Asperger vocational 7 results

Asperger's vocational 2 results

Pervasive Developmental Disorder vocational 5 results

132 results total, imported into EndNote, de-duplicated, left with **114 unique results.**

Another librarian searched several additional databases after these searches above were done:

**Canadian Business & Current Affairs Database (CBCA)**

Platform: ProQuest

Conducted: August 22, 2017

NOTE: The previous search was conducted of CBCA Education database, however this is no longer available to search as a sub-collection and so the update was performed on the entire CBCA database.

all(Autis* OR Asperger* OR ASD* OR "Pervasive Development Disorder*" OR "Pervasive Child Development Disorder*" OR "Pervasive developmental disorder*" OR "PDD-NOS" OR Rett* OR "Childhood Disintegrative Disorder*" OR "Heller's syndrome" OR "Heller syndrome") AND all(Employ* OR "pre-employ*" OR Work* OR Vocation* OR occupation* OR trade* OR career* OR job* OR rehabilitat* OR skill*)

Limits applied

Databases: Canadian Business & Current Affairs Database

Limited by:

Date: After 01 January 2008

Source type:

4 types searched: Books, Dissertations & Theses, Reports, Scholarly Journals

RESULTS: 103

**Canadian Research Index**

Platform: ProQuest

Conducted: August 22, 2017

all(Autis* OR Asperger* OR ASD* OR "Pervasive Development Disorder*" OR "Pervasive Child Development Disorder*" OR "Pervasive developmental disorder*" OR "PDD-NOS" OR Rett* OR "Childhood Disintegrative Disorder*" OR "Heller's syndrome" OR "Heller syndrome") AND all(Employ* OR "pre-employ*" OR Work* OR Vocation* OR occupation* OR trade* OR career* OR job* OR rehabilitat* OR skill*)

Limits applied

Databases: Canadian Research Index

Limited by:

Date: After 01 January 2008

RESULTS: 2

**FRANCIS**

abstract.\*:(Autis* OR Asperger* OR ASD* OR "Pervasive Development Disorder*" OR "Pervasive Child Development Disorder*" OR "Pervasive developmental disorder*" OR "PDD-NOS" OR Rett* OR "Childhood Disintegrative Disorder*" OR "Heller's syndrome" OR "Heller syndrome") AND abstract.\*:(Employ* OR "pre-employ*" OR Work* OR Vocation* OR occupation* OR trade* OR career* OR job* OR rehabilitat* OR skill*) AND abstract.\*:(treatment* OR interv* OR model* OR program* OR practice* OR instruc* OR train* OR service* OR "supported employ*") AND year.raw:[2008 TO 2017]

RESULTS: 771
